# Validation of Three Early Ejaculation Diagnostic Tools: A Composite Measure Is Accurate and More Adequate for Diagnosis by Updated Diagnostic Criteria

**DOI:** 10.1371/journal.pone.0077676

**Published:** 2013-10-15

**Authors:** Patrick Jern, Juhana Piha, Pekka Santtila

**Affiliations:** 1 Queensland Institute of Medical Research, Brisbane, Australia; 2 Department of Psychology and Logopedics, Abo Akademi University, Turku, Finland; 3 Medical Centre Mehilainen, Turku, Finland; Université Catholique de Louvain, Belgium

## Abstract

**Purpose:**

To validate three early ejaculation diagnostic tools, and propose a new tool for diagnosis in line with proposed changes to diagnostic criteria. Significant changes to diagnostic criteria are expected in the near future. Available screening tools do not necessarily reflect proposed changes.

**Materials and Methods:**

Data from 148 diagnosed early ejaculation patients (*M*_*age*_ = 42.8) and 892 controls (*M*_*age*_ = 33.1 years) from a population-based sample were used. Participants responded to three different questionnaires (*Premature Ejaculation Profile; Premature Ejaculation Diagnostic Tool; Multiple Indicators of Premature Ejaculation*). Stopwatch measured ejaculation latency times were collected from a subsample of early ejaculation patients. We used two types of responses to the questionnaires depending on the treatment status of the patients 1) responses regarding the situation before starting pharmacological treatment and 2) responses regarding current situation. Logistic regressions and Receiver Operating Characteristics were used to assess ability of both the instruments and individual items to differentiate between patients and controls.

**Results:**

All instruments had very good precision (Areas under the Curve ranging from .93-.98). A new five-item instrument (named *CHecklist for Early Ejaculation Symptoms* – CHEES) consisting of high-performance variables selected from the three instruments had validity (Nagelkerke *R* ^*2*^ range .51-.79 for backwards/forwards logistic regression) equal to or slightly better than any individual instrument (i.e., had slightly higher validity statistics, but these differences did not achieve statistical significance). Importantly, however, this instrument was more in line with proposed changes to diagnostic criteria.

**Conclusions:**

All three screening tools had good validity. A new 5-item diagnostic tool (CHEES) based on the three instruments had equal or somewhat more favorable validity statistics compared to the other three tools, but is more in line with recently proposed diagnostic criteria.

## Introduction

Recently, sustained efforts have been made to address problems that affect diagnosis of early ejaculation (EE), chief among these is the lack of a universally agreed-upon definition and valid screening tools that capture diagnostically relevant features [1]. Concerns have been raised that definitions and diagnostic criteria that rely on subjective indicators of EE (e.g. ejaculation-related distress) inflate prevalence estimates and result in over-diagnosis in normally-functioning men [2]. Thus, the International Society for Sexual Medicine (ISSM) has proposed a definition which, in addition to patient-reported outcomes (ejaculatory control, negative consequences), takes ejaculation latency time (ELT) “prior to or within about one minute” (*p*. 2949) into account [3]. The American Psychiatric Association (APA) has since followed suit, and the proposed criteria for EE in the upcoming 5th Edition of the *Diagnostic and Statistical Manual of Mental Disorders* (DSM-5, proposed EE diagnostic criteria can be seen at http://www.dsm5.org/Lists/ProposedRevision/DispForm.aspx?ID=174) now include ELT (“approximately one minute”) during partnered sexual activity. The pursuit of formulating an accurate definition of EE has also been hampered by the fact that its causal mechanisms are poorly understood, although current evidence suggests that various neurotransmitters (including serotonin and dopamine) as well as genetic factors play a role in EE etiology (for a more detailed review of the pathophysiology of EE, please see 1).

Various methods of definition and diagnosis have been suggested in the literature, with EE traditionally being diagnosed by a physician in accordance with DSM-IV (the DSM-5’s predecessor) or the World Health Organization’s *International Classification of Diseases* (ICD-10) criteria [1,2]. However, clinician-based diagnoses have been criticized for being imprecise [3]. To address this, a common measure in recent studies has been the stop-watch measured intra-vaginal ELT (IELT, [4]), which typically generates population-wide prevalence estimates of less than 2% [5], compared to ~30% when using patient-reported outcome-based definitions [6]. While this measure has some appealing properties, not least statistically as it forms a continuous, linear variable, it alone does not capture all aspects considered diagnostically relevant by the APA and the ISSM. Furthermore, given that multiple stop-watch timed measures must be collected in order to estimate a mean ELT, it is also impractical for clinical purposes. However, self-reported and stop-watch measures of ELTs are highly correlated [7], as we shall also show in the present study.

There are a number of questionnaires available for EE diagnosis, of which we shall focus on three recently published ones here (see Supporting Information): the Premature Ejaculation Diagnostic Tool (PEDT, [8]), the Premature Ejaculation Profile (PEP, [9]), and finally, *Multiple Indicators of Premature Ejaculation* (MIPE [10], note that MIPE is unnamed in the aforementioned reference), the latter a modified version of an unpublished questionnaire developed by Grenier and Byers [11]. Relatively few studies have been conducted to assess and validate these questionnaires, but reports suggest satisfactory reliability and validity for all three instruments. However, unlike MIPE, neither PEDT nor PEP has items inquiring about ejaculatory latency (although both have been compared to self-report IELT data to some extent, [9,12]). The purpose of the present study was to:

1. Validate these three questionnaires using responses from both EE patients and population controls to compare their validity; and
2. Investigate whether a more valid measure of EE could be created by selecting variables from all three questionnaires (i.e. variables that best differentiated between patients and controls).

Some of the patients who participated in the present study had received or were currently receiving pharmacological treatment for their EE problems. They reported on their ejaculatory functioning both as it was currently, and how it was prior to the commencement of the treatment. We expected using the pre-medication values for the patients to result in clearer differences between patients and controls as some of the patients’ values were expected to have been improved as a result of the treatment.

## Materials and Methods

### Ethics Statement

 The research plan was approved by the Ethics Committee of the Abo Akademi University (data collection from control group) and the Ethics Committee of the Hospital District of South-West Finland (data collection from the EE patients). Written informed consent was obtained from all participants.

### Participants

Analyses were based on the responses of 148 men diagnosed with EE (hereafter: “EE patients”) (*M*_*age*_ = 42.8, *SD* = 10.7) and 892 controls (*M*_*age*_ = 33.1, *SD* = 4.8, *p* < .0001), who had information available on the instruments used (age was not associated with any of the EE measures when analyzed separately for patients and controls in line with previous research [13]). The control group was drawn from a population-based sample of Finnish twins and siblings of twins. 

Data were collected from both EE patients and controls in 2012. Prior to data collection, we obtained updated postal addresses from the Central Population Registry of Finland for all. Data were collected through a secure online connection following inquiries by postal mail with an invitation to participate in the study. All aspects of the study, including its voluntary nature, were readily explained in the invitation letter. Participants were instructed to log onto the questionnaire using a personal eight-digit code. If a potential participant had not responded after two weeks from the initial contact, a reminder letter was sent. Participants were eligible to participate in a lottery, in which they could win one of three gift vouchers to a national travel agency (one lottery for EE patients and one for the controls, both involving 2x€500 and 1x€1000 gift cards). 

The group of EE patients was invited to participate in a stopwatch study, in which participants were to record data regarding ejaculatory latency with a stopwatch. Participants interested in this study were sent an instruction letter and a stop watch by postal mail, and given the option to complete this part of the data collection using either a secure internet diary or a paper diary which was to be returned by mail upon completion. Participants were requested to record ELT data from at least five, and a maximum of ten, sexual intercourses (anal or vaginal). In order to motivate participation, participants were reimbursed with a nominal sum for their participation (€10 per intercourse, for a maximal reimbursement of €100/participant). 24 participants eventually submitted stopwatch measured ELT data.

In total, we contacted 419 individuals who had sought treatment for EE at two clinics in Finland (EE patients). They were identified from the second author’s patient registry. In total, 178 of those we approached responded (four did not wish to participate, and 26 did not complete necessary parts for the present study), and an additional four invitation letters were unable to be delivered, suggesting that these individuals had moved or died shortly before the contact attempt, resulting in a final response rate of 43.3% for the EE patients. 

Regarding the control group, 2559 individuals were contacted by postal mail. In total, 1054 individuals completed the questionnaire. A total of 1173 responded and of these, 124 did not wish to participate, or did not complete the necessary parts of the questionnaire. Also, five individuals could not be reached (invitation letters sent to these were returned to the researchers presumably because these individuals had e.g. moved or died), resulting in a final response rate of 46% for the control group (control group *n* differs somewhat due to using one individual per family in statistical analyses, to control for between-subjects dependence due to controls being derived from a sample of twins and siblings of twins).

### Instruments

Prior to the study, all instruments were back-translated into Finnish. Note that we used two different responses from the patients depending on their treatment status: 1) regarding the situation prior to commencing medication (completed by those who had received or were currently receiving medication for EE), and 2) regarding the current situation (completed by all, including controls). 

#### Premature Ejaculation Profile

The PEP consists of four items that participants respond to on a 5-grade Likert scale ranging from 1-5. Lower scores indicate more severe EE-related problems. PEP has been shown to have good psychometric properties (test-retest-reliabilities ranging from .66 to .83 across items in two different samples, [9]). In the present study, it was found to have good reliability with Cronbach’s α being .73 for patients before treatment, and .85 at present (α controls = .82).

#### Premature Ejaculation Diagnostic Tool

PEDT consists of five items responded to on a 5-grade Likert scale from 1-5, with higher values indicating more EE problems. It has also been shown to have good psychometric properties (e.g. Cronbach’s α = .77, [12]). In the present study, Cronbach’s α for patients was .75 before medical treatment (.89 at present), and .88 for controls.

#### Multiple Indicators of Premature Ejaculation

MIPE consists of seven items, of which five are responded to on 5-grade, and two on three-grade, scales. MIPE has not been subject to case-controlled validity analyses, but extensive confirmatory factor analyses suggest good reliability [10]. In the present study, Cronbach’s α for EE patients was .59 before medical treatment (.84 at present) and .77 for controls. The item measuring self-reported ELT (including response options) is found in the Appendix.

#### Stop-Watch Measured Ejaculation Latency Time

We collected stop-watch measured ejaculation latency time data from 24 EE patients (each providing 3-10 recoded ELTs, *M* = 8.00 observations, *SD* = 2.11). These data were used to compute geometric mean ELTs [14], based upon which ELT values were imputed for the other EE patients using information from PEP, PEDT and MIPE scores in the expectation maximization procedure in SPSS 21.0.

### Statistical analyses

To control for dependence between observations of men belonging to the same family, we randomly excluded 135 men whose brothers also participated, so that only one person per family was represented. We used independent and paired samples *t*-tests to compare means on the ejaculatory function measures between EE patients and controls, and of EE patients in pre-medication and current settings. Pearson correlations were used to investigate the co-variation of different variables. We used logistic regression analyses (Forward and Backward selection procedures using the likelihood ratio criterion with a p-value of .05 for entry and .10 for removal from the model; both types of selection procedures were used to increase the robustness of the selection procedure) to identify items from among all items of the three EE instruments that best differentiated between patients and controls. These analyses were done separately using pre-medication and current scores for the EE patients. Items that remained in the final models using both Forward and Backward selection were retained for further analyses. We used Receiver Operating Characteristics (ROC) analyses to assess the ability of different instruments to differentiate between the EE patients and controls using the Area Under Curve (AUC) value. 

## Results

### Ejaculatory functioning in EE patients and controls

The values on the different instruments correlated highly for both EE patients (both pre-medication and currently) and controls, indicating that all three instruments measured the same construct (Table 1). EE patients’ ejaculatory functioning improved from pre-medication to the current situation, and the ejaculatory functioning measured in the control group was better both compared to the EE patients’ pre-medication measurement as well as compared to their current values (Table 2). This was the case independent of the instrument used. 

**Table 1:** Correlations between measures of ejaculatory functioning in patients (pre-medication and current) and controls.

	**Patients**				**Controls**	
	*Pre-medication*		*Current*			
	PEP	PEDT	PEP	PEDT	PEP	PEDT
PEDT	-.88		-.77		-.82	
MIPE	-.59	.67	-.50	.57	-.80	.86

Note. PEP = Premature Ejaculation Profile [9]; PEDT = Premature Ejaculation Diagnostic Tool [8]; MIPE = Multiple Indicators of Premature Ejaculation [10]. For PEP, higher values indicate better ejaculatory functioning; for PEDT and MIPE, lower values indicate better ejaculatory functioning. All correlations were significant at the *p* < .001 level.

**Table 2:** Ejaculatory functioning in patients (pre-medication and current) and controls.

	**Patients**				**Patients**				**Patients vs.Controls**			
	*Before medication*		*Current (incl. individuals both on and off medication)*		*Before medication > Current*		*Controls*		*Cf. to Before medication*		*Cf. to Current*	
	*M*	*SD*	*M*	*SD*	Mean difference (95% C.I.)	*t^a^*	*M*	*SD*	Mean difference (95% C.I.)	*t^b^*	Mean difference (95% C.I.)	*t^b^*
PEP	7.13	2.25	11.1	3.85	-4.04 (-4.74…-3.33)	11.33	16.31	2.94	9.17 (8.65…9.69)	34.7	5.21 (4.67…5.74)	18.99
PEDT	21.04	3.14	16.82	5.04	4.31 (3.44…5.20)	9.69	9.14	3.97	-11.90 (-12.60…-11.20)	33.26	-7.68 (-8.40…-6.96)	20.89
MIPE	24.78	2.96	21.33	4.72	3.48 (2.68…4.29)	8.54	13.71	3.74	-11.07 (-11.73…-10.41)	32.85	-7.63 (-8.31..-6.95)	18.67

Note. ^a^ Paired-samples *t*-test comparing the values of the patients pre-medication and currently (*n* = 135). ^b^ Independent samples *t*-tests comparing controls (*n* = 892) to patients’ pre-medication (*n* = 135) and current values (*n* = 148). PEP = Premature Ejaculation Profile [9]; PEDT = Premature Ejaculation Diagnostic Tool [8]; MIPE = Multiple Indicators of Premature Ejaculation [10]. For PEP, higher values indicate better ejaculatory functioning; for PEDT and MIPE, lower values indicate better ejaculatory functioning. All differences were significant at the *p* < .001 level. *C*.*I*. = Confidence interval.

The geometric mean stop-watch measured ELT for EE patients was 132s (*SD* = 93s, 22.9% <1 min). Note that stopwatch ELTs were imputed for most EE patients as described in the Method section, and that these are inclusive of individuals currently on EE medication. Before imputation, the geometric mean stop-watch measured ELTs was 123s (*SD* = 133s, 50% <1 min). Stop-watch measured ELT had high correlations with both the different ejaculatory function instruments (current reports were used here as the stop-watch measurements were also done currently; correlations without imputed data in brackets): (PEP *r* = .78, *p* < .001 [*r* = .51, *p* < .011]; PEDT *r* = -.81, *p* < .001 [*r* = -.60, *p* < .002]; MIPE *r* = -.73, *p* < .001 [*r* = -.49, *p* < .016]) as well as with the self-reported ELT (Self-reported ELT *r* = -.71 *p* < .001 [*r* = -.57, *p* < .004]). *n* was 148 for analyses using imputed data and 24 for analyses using unimputed data.

### Ability of different ejaculatory function instruments to differentiate between EE patients and controls

Next, we investigated how well the different instruments as well as the self-reported ELT were able to differentiate between EE patients and controls using ROC analysis (Table 3). The *AUC* values were consistently very high and did not differ between the three instruments. As expected, the values were in each instance higher (as indicated by the non-overlap of the confidence intervals) when pre-medication (vs. current) values were used for the EE patients. Using only the self-reported ELT also resulted in good differentiation between the groups even if it was worse compared to MIPE and PEP when pre-medication values were used for EE patients. 

**Table 3:** Ability of ejaculatory function instruments and self-reported ejaculation latency time to differentiate between patients and controls.

	*AUC*	*SE of AUC*	95% C.I.	
			Lower Bound	Upper Bound
	**Pre-medication values for patients**			
PEP	0.98	0.00	0.97	0.99
PEDT	0.97	0.01	0.96	0.98
MIPE	0.98	0.00	0.97	0.98
Self-reported ejaculation latency time ^a^	0.94	0.01	0.92	0.96
	**Current values for patients**			
PEP	0.84	0.02	0.80	0.89
PEDT	0.87	0.02	0.84	0.91
MIPE	0.88	0.02	0.85	0.91
Self-reported ejaculation latency time ^a^	0.83	0.02	0.79	0.86

Note. Patients (pre-medication values: *n* = 135, current values: n = 148) and controls (*n* = 892). *C*.*I*. = confidence interval. PEP = Premature Ejaculation Profile [9]; PEDT = Premature Ejaculation Diagnostic Tool [8]; MIPE = Multiple Indicators of Premature Ejaculation [10]. ^a^ The following item from MIPE: *On* *average, during* *intercourse, how* *much* *time* *elapses* *between* *when* *you* *first* *enter* *your* *partner* (*vaginally or anally*) *with* *your* *penis and when* *you* *first* *ejaculate*? 0 = I usually do not ejaculate, 1 = More than 10 min., 2 = Between 5 and 10 min., 3 = Between 1 and 5 min., 4 = Less than 1 min. *AUC* = Area under the Curve from the Receiver Operating Characteristics analysis of diagnostic efficacy of the different measures (1.00 indicates perfect differentiation between groups whereas 0.50 indicates chance level differentiation between groups).

### Identifying individual items best able to differentiate between EE patients and controls

Next, we used logistic regression analyses to identify from among the items of all three instruments those best able to differentiate between EE patients and controls. Both forward and backward stepwise (likelihood ratio) logistic regressions were run; items that were selected by both procedures into the final model were selected for further analyses. Table 4 shows the final models. All models had high Nagelkerke *R* ^*2*^. As expected, models using pre-medication values differentiated between patients and controls better than models based on post-medication values. Using the pre-medication values, five variables were included in both the final models using forward and backward selection. Using current values, five variables (not exactly the same) were also included in both models. 

**Table 4:** Final models from logistic regression analyses using all items from the three ejaculatory function instruments to differentiate between patients and controls.

	*B*	*SE*	*Wald χ2*	Odds ratio (95% C.I.)	Model	Nagelkerke *R* ^*2*^
	Pre-medication values for patients					
*Forward (likelihood ratio)*					575.02, *p* < .000001	0.79
**PEP_control**	-1.23	0.31	15.62***	0.29 (0.16…0.54)		
**PEP_relationshipproblems**	-0.41	0.19	4.82*	0.67 (0.46…0.96)		
**PEDT_littlestimulation**	-0.47	0.18	6.57**	0.63 (0.44…0.90)		
**PEDT_frustrated**	0.69	0.24	7.90**	1.99 (1.23…3.21)		
**MIPE_elt**	1.10	0.33	11.36***	3.01 (1.59…5.71)		
MIPE_worrying	0.52	0.25	4.15*	1.68 (1.02…2.76)		
*Backward (likelihood ratio)*					574.68, *p* < .000001	0.79
**PEP_control**	-1.19	0.31	14.51***	0.30 (0.17…0.56)		
**PEP_relationshipproblems**	-0.43	0.19	5.39*	0.65 (0.45…0.94)		
**PEDT_littlestimulation**	-0.50	0.19	7.27**	0.61 (0.42…0.87)		
**PEDT_frustrated**	0.78	0.23	11.51***	2.17 (1.39…3.40)		
**MIPE_elt**	1.15	0.33	12.44***	3.15 (1.66…5.95)		
MIPE_toosoon	0.79	0.39	4.05*	2.20 (1.02…4.76)		
	Current values for patients					
*Forward (likelihood ratio)*					348.32, *p* < .00001	0.51
**PEP_control**	0.50	0.16	9.785**	1.65 (1.21…2.26)		
**PEDT_frustrated**	0.36	0.16	5.244*	1.44 (1.05…1.95)		
**PEDT_concerned**	0.42	0.14	8.662**	1.52 (1.15…2.01)		
**MIPE_elt**	0.85	0.19	19.834***	2.33 (1.60…3.37)		
**MIPE_worrying**	0.67	0.14	22.115***	1.96 (1.48…2.60)		
*Backward (likelihood ratio)*					358.01, *p* < .000001	0.52
**PEP_control**	0.34	0.18	3.77	1.41 (1.00…2.00)		
PEP_satisfaction	0.27	0.16	2.77	1.31 (0.95…1.80)		
PEP_relationshipproblems	-0.36	0.16	5.06*	0.70 (0.52…0.96)		
PEDT_littlestimulation	-0.24	0.14	2.98	0.79 (0.60…1.03)		
**PEDT_frustrated**	0.47	0.17	7.89**	1.60 (1.15…2.23)		
**PEDT_concerned**	0.33	0.15	4.55*	1.39 (1.03…1.88)		
**MIPE_elt**	0.88	0.20	20.00***	2.40 (1.64…3.52)		
**MIPE_worrying**	0.67	0.14	21.25***	1.95 (1.47…2.58)		

Note. Items that were included in the final model both using forward and backward selection procedures have been indicated with bold typeface. PEP = Premature Ejaculation Profile [9]; PEDT = Premature Ejaculation Diagnostic Tool [8]; MIPE = Multiple Indicators of Premature Ejaculation [10]. SE = standard error. Please see Supplementary Information for complete descriptions of the variables in each questionnaire.

* *p* < .05, **p < .01, ***p < .001

### Using sum scores based on the selected individual items to differentiate between EE patients and controls

Next, we calculated a sum score (the PEP items were reversed prior to this) based on the pre-medication and current items that were included in both the forward and backward logistic regressions, respectively. Both variables had significantly higher values among the EE patients (based on variables from pre-medication logistic regressions *M* = 20.42, *SD* = 2.83; Based on variables from the current logistic regressions *M* = 18.45, *SD* = 4.29) compared to controls (Pre-medication *M* = 9.92, *SD* = 3.36; Current *M* = 10.50, *SD* = 3.87). Both the Pre-medication (*t*[1025] = 34.53, *p* < .001) and the Current (*t*[1038] = 22.79, *p* < .001) comparisons were significant. Both variables also resulted in very good differentiation between the EE patients and the controls in an ROC analysis: Pre-medication: AUC = 0.98, *SE* = 0.00, 95%*C.I*: 0.97-0.98; Current: AUC = 0.90, *SE* = 0.01, 95%*C.I*: 0.87-0.93. The confidence intervals indicate that the pre-medication values resulted again, as expected, in better differentiation. Cronbach’s alpha reliabilites ranged from .64-.86. The new instrument had also higher correlations with both imputed and unimputed (*r* = -.84, *p* < .001) stop-watch measured ELTs compared to the other scales. 

### Performance of different cut-off scores

Table 5 shows (using the pre-medication values for the patients) the ability of the different instruments to identify EE patients. As the instruments are for screening purposes, we want to keep the sensitivity of the instrument high, that is, we do not want to lose potential EE sufferers by using too high a threshold. If we, for example, want to identify 90% of EE patients, the cut-off scores are the following (% of controls wrongly identified as patients with these cut-offs in parentheses: PEP: 10 (6%), PEDT: 17 (6%), MIPE: 21 (6%), New: 17 (5%). The differences are small with the new instrument keeping the false positive rate at its lowest. 

**Table 5:** Ability of three different diagnostic instruments for early ejaculation to separate between patients and controls.

	**PEP**				**PEDT**				**MIPE**				**CHEES**			
	*False positives*	*Hits*			*False positives*	*Hits*			*False positives*	*Hits*			*False positives*	*Hits*		
Score	1-Specificity	Sensitivity	Control	Case	1-Specificity	Sensitivity	Control	Case	1-Specificity	Sensitivity	Control	Case	1-Specificity	Sensitivity	Control	Case
4	0.3	100.0	3	15												
5	0.7	88.9	3	21	100.0		142						100.0		1	
6	1.1	73.3	4	22	84.1		122						99.9		68	
7	1.6	57.0	4	24	70.4		121		100.0		1		92.3		144	
8	2.1	39.3	5	17	56.8		97		99.9		3		76.1		154	
9	3.8	26.7	15	19	46.0		93		99.6		43		58.9		145	
10	5.6	12.6	16	8	35.5		66		94.7		112		42.6		86	
11	7.8	6.7	20	4	28.1	0.7	54	1	82.2		134		33.0		82	
12	10.3	3.7	22	2	22.1	1.5	49	1	67.2		123		23.8	1.5	58	2
13	14.7	2.2	39		16.6	2.2	36	1	53.4		110		17.3	1.5	42	
14	19.7	2.2	45	2	12.6	3.0	25	1	41.0		76		12.6	2.2	26	1
15	30.0	0.7	92	1	9.8	4.4	19	2	32.5		68		9.6	5.2	21	4
16	44.7		131		7.6	7.4	12	4	24.9		46		7.3	6.7	18	2
17	58.3		121		6.3	14.1	11	9	19.7		38		5.3	14.8	7	11
18	77.1		168		5.0	21.5	11	10	15.5	1.5	40	2	4.5	24.4	11	13
19	91.1		125		3.8	31.1	9	13	11.0	6.7	30	7	3.3	38.5	9	19
20	100.0		79		2.8	42.2	5	15	7.6	9.6	18	4	2.2	51.9	8	18
21					2.2	49.6	3	10	5.6	14.1	8	6	1.3	61.5	1	13
22					1.9	61.5	5	16	4.7	20.0	8	8	1.2	69.6	3	11
23					1.3	72.6	6	15	3.8	34.1	11	19	0.9	85.2	5	21
24					0.7	84.4	5	16	2.6	46.7	6	17	0.3	94.8	2	13
25					0.1	100.0	1	21	1.9	54.8	7	11	0.1	100.0	1	7
26									1.1	68.9	4	19				
27									0.7	80.0	3	15				
28									0.3	89.6	1	13				
29									0.2	97.0		10				
30									0.2	99.3		3				

Note. PEP = Premature Ejaculation Profile [9]; PEDT = Premature Ejaculation Diagnostic Tool [8]; MIPE = Multiple Indicators of Premature Ejaculation [10]; CHEES = CHecklist for Early Ejaculation Symptoms (proposed new diagnostic instrument). For PEP, higher values indicate better ejaculatory functioning; for PEDT and MIPE, lower values indicate better ejaculatory functioning.

## Discussion

Here, we show that all three tested instruments (PEP, PEDT, MIPE) are valid tools to separate between EE patients and controls, all having very good specificity and sensitivity to distinguish between patients and controls. In addition, an important characteristic of any good screening tool is – besides the obvious ability to discriminate between untreated patients and controls – to detect change in the trait it is designed to measure. Again, all three screening tools were able to detect significant change between pre-medication and current status in the group of EE patients. However, a combination measure featuring two items each from PEP and PEDT, and one item from MIPE covers factors considered important for diagnosis outlined in recent proposals for EE diagnostic criteria [1,2] while maintaining equally good validity and precision (even somewhat higher validity scores). Furthermore, the new instrument also had higher correlations with both imputed and non-imputed stopwatch measures of ELT compared to the other three instruments. We propose the name *Checklist for Early Ejaculation Symptoms* (CHEES) for this brief, composite instrument, which is presented with proposed diagnostic cutoff scores in Table 6.

**Table 6:** Proposed new diagnostic screening tool: CHecklist for Early Ejaculation Symptoms (CHEES; questions should be responded to considering the past 6 months).

**Question**	**Response options**
*1. Over the past six months, was your control over ejaculation during sexual intercourse*: ^a, †^	1: Very good
	2: Good
	3: Fair
	4: Poor
	5: Very Poor
*2. To what extent does how fast you ejaculate (come*)* during sexual (vaginal*)* intercourse cause difficulty in your relationship with your partner*? ^a, †^	1: Not at all
	2: A little bit
	3: Moderately
	4: Quite a bit
	5: Extremely
*3. Do you ejaculate with very little stimulation*? ^b^	1: Almost never or never
	2: Less than half the time
	3: About half the time
	4: More than half the time
	5: Almost always or always
*4. Do you feel frustrated because of ejaculating before you want to*? ^b^	1: Not at all
	2: Slightly
	3: Moderately
	4: Very
	5: Extremely
*5. On average, during intercourse, how much time elapses between when you first enter your partner (vaginally or anally*)* with your penis and when you first ejaculate*? ^c^	1: I usually do not ejaculate
	2: More than 10 min.
	3: Between 5 and 10 min.
	4: Between 1 and 5 min.
	5: Less than 1 min.

Scoring legend and proposed diagnostic cut-offs:

21-25: Strongly indicative of fulfilling diagnostic criteria for EE (correctly identifies 44% of patients as EE sufferers and incorrectly identifies 1% of controls as EE sufferers)17-20: Indicative of EE (correctly identifies 90% of patients as EE sufferers and incorrectly identifies 5% of controls as EE sufferers)5-16: Low probability of EE

Note. A score of at least 5 on the final question could be used as a prerequisite in order to strictly comply with updated DSM-5 and ISSM criteria [1,2]. Note that this questionnaire does not consider differential diagnoses (e.g. opioid withdrawal symptoms, multiple sclerosis), therefore a clinical interview should also be conducted to rule out such factors. ^a^ adapted from the Premature Ejaculation Profile [9], ^b^ adapted from the Premature Ejaculation Diagnostic Tool [8], ^c^ adapted from the Multiple Indicators of Premature Ejaculation [10]. † order of response options has been reversed from the original questionnaire. EE = early ejaculation.

 In order to comply with ISSM and DSM-5 definitions, the ejaculatory latency should ideally be one minute or less for diagnosis. An ELT latency cut-off has been considered necessary for diagnosis because definitions that rely solely on subjective perceptions tend to inflate prevalence rates of EE, and unnecessarily label normal ejaculatory function as pathological. It should be noted, however, that some confusion remains regarding the optimal ELT cutoff for EE diagnosis. As noted previously, both the ISSM and the DSM-5 definitions suggest a cutoff of “about” one minute (or less), allowing for some flexibility for diagnosing physicians. Hence, greater emphasis could be placed on the fifth question of the newly proposed instrument, in order to avoid that individuals with comparatively long ejaculatory latencies are misdiagnosed. Indeed, about 30% of men express subjective concerns regarding their ejaculatory function [6], whereas only 1-2% of men in the general population have consistent ELTs of less than a minute [5,13]. Four different “subtypes” of EE have been proposed in the literature, with two of these (“natural variable premature ejaculation” and “premature-like ejaculatory dysfunction”) being described as temporary, circumstantial (i.e., non-chronic) problems or psychological misrepresentations of normal ejaculatory function [15]. On the other hand, it can be argued that a short ejaculatory latency can, ultimately, only be considered to be dysfunctional if it occurs before penetration (as the ultimate function of ejaculation is to impregnate a female, and there is currently no evidence to support that conception is less likely if the ELT is short). As EE is treated to improve recreational, rather than procreational, sexual activity, it is questionable whether it is defensible to give greater weight to ELT over patient-reported outcomes in EE diagnosis [16], however it is arguably problematic to prescribe EE medication that can potentially be harmful to an individual who presents subjective complaint of EE but has a relatively long ejaculatory latency. Hence, we suggest that patients should score at least 4 (between 1 and 5 minutes) on the fifth question in Table 5. Clinicians are also advised to inquire about ELT separately when meeting with patients, and pay special attention to other EE indicators in borderline cases (i.e. if the patient scores a 4 on the aforementioned questionnaire item).

 In the literature, (I)ELT has been proposed as a necessary parameter for accurate measurement of EE [1,3-5]. However, the (I)ELT as a measure has some drawbacks: firstly, it is costly and difficult to manage in large-scale studies, although some clinical trials using stopwatch measured ELT data have amassed study samples close to 2,000 individuals (e.g. [17]). Concerns have also been raised that it is unsuitable in the clinical setting, since some individuals may be reluctant to record the duration of sexual intercourse with a stopwatch, and many clinicians consider stopwatch measurement of ELT impractical [18]. These concerns are supported by the results of the present study, as about 5/6 participants who volunteered for a survey study chose not to participate in a study involving actual recording of stopwatch data, even though they received financial reimbursement for doing so. This, in addition to research findings suggesting that self-estimated ejaculatory latencies are accurate reflections of stopwatch measured ejaculatory latencies in general, underlines the need for accurate diagnostic tools that are simple to use in both clinical and research settings.

It is important to note that the control group in the present study is a population-based sample (i.e. not a selected group of non-affected individuals, but a group inclusive also of men who display EE symptoms). In the light of this, the sensitivity and specificity of the measures, especially the newly proposed measure, would likely be even more convincing had we used a non-affected control group.

Limitations to be considered for this study is the relatively low number of participants who provided stopwatch data (24 patients), as well as retrospective measures of ejaculatory function prior to use of medication in the patient group. Data were not recorded in laboratory settings but by the participants themselves, and this may be a source of bias. The response rate was somewhat low, but fully comparable to other sexuality-related surveys in which large cohorts has been approached at a single occasion without pre-screening [13,19,20]. Furthermore, all EE patients had been diagnosed with premature ejaculation by a physician specializing in sexual medicine.

## Conclusions

In conclusion, three commonly used diagnostic tools for EE were shown to have good reliability and validity. A composite measure can be derived from these tools to form a new, valid tool which is adequate considering proposed changes to diagnostic criteria. Studies assessing the newly proposed questionnaire in independent samples are welcome.

## Supporting Information

Table S1 **Three instruments for diagnosis and measurement of early ejaculation.** *Note*. All variables have been converted to a 1-5 or 1-3 scale. PEP = Premature Ejaculation Profile ^9^; PEDT = Premature Ejaculation Diagnostic Tool ^8^; MIPE = Multiple Indicators of Premature Ejaculation ^10^. For PEP, higher scores indicate better function; for MIPE and PEDT, lower scores indicate better function. Items in boldface type have the greatest effect sizes and constitute the new proposed diagnostic tool.
(DOCX)
