# Supplementary material for: Validation of Three Early Ejaculation Diagnostic Tools: A Composite Measure Is Accurate and More Adequate for Diagnosis by Updated Diagnostic Criteria
Source: PLoS One. 2013 Oct 15;8(10):e77676. doi: 10.1371/journal.pone.0077676 (PMC3797135; doi:10.1371/journal.pone.0077676)
Supplement: Table S1 — Three instruments for diagnosis and measurement of early ejaculation. Note. All variables have been converted to a 1-5 or 1-3 scale. PEP = Premature Ejaculation Profile 9; PEDT = Premature Ejaculation Diagnostic Tool 8; MIPE = Multiple Indicators of Premature Ejaculation 10. For PEP, higher scores indicate better function; for MIPE and PEDT, lower scores indicate better function. Items in boldface type have the greatest effect sizes and constitute the new proposed diagnostic tool. (DOCX) [file pone.0077676.s001.docx]

**Table S1 – Three instruments for diagnosis and measurement of early ejaculation**

| Instrument | Variable name | Question | Response options and scores |
| --- | --- | --- | --- |
| PEP (past 4 weeks) | **PEP_control** | ***Over the past month, was your control over ejaculation during sexual intercourse:*** | **1: Very poor** |
|  |  |  | **2: Poor** |
|  |  |  | **3: Fair** |
|  |  |  | **4: Good** |
|  |  |  | **5: Very good** |
|  | PEP_satisfaction | *Over the past month, was your satisfaction with sexual intercourse:* | 1: Very poor |
|  |  |  | 2: Poor |
|  |  |  | 3: Fair |
|  |  |  | 4: Good |
|  |  |  | 5: Very good |
|  | PEP_distress | *How distressed are you by how fast you ejaculate (come) during sexual (vaginal) intercourse?* | 1: Extremely |
|  |  |  | 2: Quite a bit |
|  |  |  | 3: Moderately |
|  |  |  | 4: A little bit |
|  |  |  | 5: Not at all |
|  | **PEP_relationshipproblems** | ***To what extent does how fast you ejaculate (come) during sexual (vaginal) intercourse cause difficulty in your relationship with your partner?*** | **1: Extremely** |
|  |  |  | **2: Quite a bit** |
|  |  |  | **3: Moderately** |
|  |  |  | **4: A little bit** |
|  |  |  | **5: Not at all** |
| PEDT (past 4 weeks) | PEDT_control | *How difficult is it for you to delay ejaculation?* | 1: Not difficult at all |
|  |  |  | 2: Somewhat difficult |
|  |  |  | 3: Moderately difficult |
|  |  |  | 4: Very difficult |
|  |  |  | 5: Extremely difficult |
|  | PEDT_tooearly | *Do you ejaculate before you want to?* | 1: Almost never or never (0%) |
|  |  |  | 2: Less than half the time (25%) |
|  |  |  | 3: About half the time (50%) |
|  |  |  | 4: More than half the time (75%) |
|  |  |  | 5: Almost always or always (100%) |
|  | **PEDT_littlestimulation** | ***Do you ejaculate with very little stimulation?*** | **1: Almost never or never (0%)** |
|  |  |  | **2: Less than half the time (25%)** |
|  |  |  | **3: About half the time (50%)** |
|  |  |  | **4: More than half the time (75%)** |
|  |  |  | **5: Almost always or always (100%)** |
|  | **PEDT_frustrated** | ***Do you feel frustrated because of ejaculating before you want to?*** | **1: Not at all** |
|  |  |  | **2: Slightly** |
|  |  |  | **3: Moderately** |
|  |  |  | **4: Very** |
|  |  |  | **5: Extremely** |
|  | PEDT_concern | *How concerned are you that your time to ejaculation leaves your partner sexually unfulfilled?* | 1: Not at all |
|  |  |  | 2: Slightly |
|  |  |  | 3: Moderately |
|  |  |  | 4: Very |
|  |  |  | 5: Extremely |
| MIPE (past 2 years) | **MIPE_elt** | ***On average, during intercourse, how much time elapses between when you first enter your partner (vaginally or anally) with your penis and when you first ejaculate?*** | **1: I usually do not ejaculate** |
|  |  |  | **2: More than 10 min.** |
|  |  |  | **3: Between 5 and 10 min.** |
|  |  |  | **4: Between 1 and 5 min.** |
|  |  |  | **5: Less than 1 min.** |
|  | MIPE_thrusts | *How many penile thrusts have you typically been able to perform before ejaculation?* | 1: I usually do not ejaculate |
|  |  |  | 2: More than 10 |
|  |  |  | 3: Between 6 and 10 |
|  |  |  | 4: Between 1 and 5 |
|  |  |  | 5: No thrusts at all |
|  | MIPE_anteportal | *In what percentage of sexual intercourse experiences do you involuntarily ejaculate before intercourse has started?* | 1: Never or rarely |
|  |  |  | 2: Less than half the time |
|  |  |  | 3: About half the time |
|  |  |  | 4: More than half the time |
|  |  |  | 5: Almost always or always |
|  | MIPE_control | *How often have you felt that you could decide when to ejaculate?* | 1: Never or rarely |
|  |  |  | 2: Less than half the time |
|  |  |  | 3: About half the time |
|  |  |  | 4: More than half the time |
|  |  |  | 5: Almost always or always |
|  | MIPE_worrying | *How often have you been worried that you would ejaculate sooner than you would like to?* | 1: Never or rarely |
|  |  |  | 2: Less than half the time |
|  |  |  | 3: About half the time |
|  |  |  | 4: More than half the time |
|  |  |  | 5: Almost always or always |
|  | MIPE_toosoon | *How often have you ejaculated sooner than you want to?* | 1: I usually do not ejaculate sooner than I want to |
|  |  |  | 2: Usually |
|  |  |  | 3: Always or almost always |
|  | MIPE_delay | How often have you tried to delay intercourse | 1: I have not usually tried to delay |
|  |  |  | 2: Usually |
|  |  |  | 3: Always or almost always |

*Note.* All variables have been converted to a 1-5 or 1-3 scale. PEP = Premature Ejaculation Profile ^9^; PEDT = Premature Ejaculation Diagnostic Tool ^8^; MIPE = Multiple Indicators of Premature Ejaculation ^10^. For PEP, higher scores indicate better function; for MIPE and PEDT, lower scores indicate better function. Items in boldface type have the greatest effect sizes and constitute the new proposed diagnostic tool.
